# Essential Oils and Their Components as Modulators of Antibiotic Activity against Gram-Negative Bacteria

**DOI:** 10.3390/medicines3030019

**Published:** 2016-07-28

**Authors:** Petruta Aelenei, Anca Miron, Adriana Trifan, Alexandra Bujor, Elvira Gille, Ana Clara Aprotosoaie

**Affiliations:** 1 Department of Pharmacognosy, Faculty of Pharmacy, University of Medicine and Pharmacy Grigore T. Popa-Iasi, Universitatii 16, Iasi 700115, Romania; petruta.aelenei@fitermanpharma.ro (P.A.); adriana.trifan@umfiasi.ro (A.T.); bujoralexandra123@gmail.com (A.B.); anaclara70@yahoo.com (A.C.A.); 2 Regulatory Affairs Department, Fiterman Pharma LLC, Pacurari Road 127, Iasi 700544, Romania; 3 Stejarul Biological Research Centre/National Institute of Research and Development for Biological Sciences, Alexandru cel Bun 6, Piatra Neamt 610004, Romania; elgille9@yahoo.com

**Keywords:** essential oils, Gram-negative bacteria, multidrug-resistance, antibiotics, synergistic interactions

## Abstract

Gram-negative bacteria cause infections that are difficult to treat due to the emergence of multidrug resistance. This review summarizes the current status of the studies investigating the capacity of essential oils and their components to modulate antibiotic activity against Gram-negative bacteria. Synergistic interactions are particularly discussed with reference to possible mechanisms by which essential oil constituents interact with antibiotics. Special emphasis is given to essential oils and volatile compounds that inhibit efflux pumps, thus reversing drug resistance in Gram-negative bacteria. In addition, indifference and antagonism between essential oils/volatile compounds and conventional antibiotics have also been reported. Overall, this literature review reveals that essential oils and their purified components enhance the efficacy of antibiotics against Gram-negative bacteria, being promising candidates for the development of new effective formulations against Gram-negative bacteria.

## 1. Introduction

Infectious diseases are the second leading cause of death worldwide, being responsible for approximately 15 million deaths each year [1]. This high mortality rate is mainly due to antibiotic resistance. Frequent and improper use of antibiotics favored the emergence and spread of single- and multidrug-resistant microbial strains [2]. In 2009, several European countries reported that 25%–50% of *Staphylococcus aureus* isolates from patients with nosocomial infections were resistant to methicillin (oxacillin). In 2012, some of these countries (Portugal and Romania) reported percentages higher than 50% for methicillin-resistant *S. aureus* isolates. Resistance to carbapenems, broad-spectrum β-lactam antibiotics, has alarmingly increased. From 2009–2012, the percentages of carbapenem-resistant *Klebsiella pneumoniae* isolates increased dramatically from very low values, such as below 1% in Romania and 1%–5% in Italy, to values of 10%–25% and 25%–50%, respectively [3]. 

Combination therapy, combining conventional antibiotics and natural products, represents a promising strategy in overcoming antibiotic resistance [4,5,6]. Such antimicrobial combinations may exhibit synergistic, additive, indifferent or antagonistic effects. Synergy, addition and antagonism occur when the overall effect is greater than, equal to and less than the sum of the individual effects, respectively; the absence of any interaction reflects indifference [7,8]. Synergy has potential therapeutic applications, as synergistic combinations have higher efficacy and lower toxicity than their components; due to a multitarget activity, such combinations may prevent the emergence of antibiotic resistance and may be effective against multidrug-resistant microbial strains [7]. Although ignored or rejected in many studies, antagonistic interactions have also important implications, as they might predict a reduction of antimicrobial efficacy [7,9]. Several methodologies have been used to assess antimicrobial interactions between conventional antibiotics and natural products. The most frequently employed is the checkerboard assay, which expresses antimicrobial interactions on the basis of the fractional inhibitory concentration index (FICI) or isobolograms [7,10]. There are different interpretations of antimicrobial interactions on the basis of FICI, for example a synergistic interaction is considered to occur if FICI ≤ 0.5 [11,12,13] or < 1 [2]. There are also different interpretations of the FICI values denoting additivity (0.5 < FICI ≤ 1, FICI = 1) [2,11,12], indifference (1 < FICI ≤ 2, 1 < FICI ≤ 4, 5 < FICI ≤ 4) [11,12,13,14] and antagonism (FICI > 1, 2 or 4) [2,11,12,13].

Most natural products have shown activity predominantly on Gram-positive bacteria [15,16,17]. Gram-negative bacteria are intrinsically more resistant to antibacterial agents than the Gram-positive ones due to an additional outer membrane acting as an effective barrier for amphipathic agents [18,19,20] and overexpression of efflux pumps responsible for innate antimicrobial resistance, such as the AcrAB-TolC efflux system [17]. Therefore, the identification of new antimicrobial agents active against Gram-negative bacteria and/or having the ability to modulate the antibiotic susceptibility of Gram-negative bacteria is of considerable importance to therapeutics.

This review summarizes data on essential oils (EOs) and pure volatile compounds that modulate antibiotic activity against Gram-negative bacteria (*Acinetobacter baumannii*, *Escherichia coli*, *Klebsiella pneumoniae*, *Pseudomonas aeruginosa*, *Enterobacter* spp., *Proteus vulgaris*, *Salmonella* spp.) with special emphasis on synergistic interactions.

## 2. Essential Oils and Volatile Compounds Modulating Antibiotic Activity against *Acinetobacter baumannii*

*Acinetobacter baumannii* is an aerobic, pleomorphic, non-motile, non-fermentative, catalase-positive and oxidase-negative coccobacillus [21,22,23]. It is responsible for skin and soft-tissue infections, being a common Gram-negative bacillus in traumatic injuries (open tibial fractures) and burns [21,24,25]. *A. baumannii* also causes bacteremia, secondary meningitis, respiratory and urinary tract infections [24], being the fourth among the most frequent etiological agents of hospital-acquired infections after *Pseudomonas aeruginosa*, *Staphylococcus aureus* and *Klebsiella pneumoniae* [22]. Its pathogenicity is due to a high ability to adhere to and form biofilms on both biotic and abiotic surfaces. Outer membrane protein A (OmpA) and fimbriae play a key role in adhesion, whereas pili and biofilm-associated protein are involved in biofilm formation. In addition, OmpA confers resistance to the serum complement and induces the apoptosis of host cells via mitochondrial dysfunction [21]. Phospholipases are important *A. baumannii* virulence factors [21,26,27]. Carbapenems (alone or in combination with an aminoglycoside), cephalosporins and β-lactam-β-lactamase inhibitor (sulbactam) combinations are commonly used in the treatment of infections caused by antibiotic-susceptible *A. baumannii* strains. *A. baumannii* has developed resistance to almost all commercial antimicrobials (β-lactams, aminoglycosides, quinolones, tetracyclines, glycylcyclines, polymyxins, sulfonamides). Both enzymatic (the production of β-lactamases, including serine- and metallo-β-lactamases responsible for carbapenem resistance, aminoglycoside-modifying enzymes) and non-enzymatic mechanisms (alterations in outer membrane proteins, efflux pumps, penicillin-binding proteins, porin channels, DNA gyrase and topoisomerase IV) are responsible for *A. baumannii* antibiotic resistance [23,28]. Multidrug-resistant (usually resistant to quinolones, cephalosporins and carbapenems), extensively drug-resistant (resistant to almost all commercially available antimicrobials, including cephalosporins, penicillins, carbapenems, aminoglycosides, fluoroquinolones, sulbactam) [29] and pan-resistant (resistant to all standard antimicrobials, except colistin) [28] strains have become a major threat to *A. baumannii* infection control. The treatment options of multidrug-resistant *A. baumannii* infections include polymyxins B and E (colistin), tigecycline and antibiotic combinations (colistin or imipenem and rifampin). Polymyxins cause severe side effects, such as nephrotoxicity and bronchoconstriction. Besides, *A. baumannii* isolates lacking susceptibility to tigecycline have been detected [28].

**Figure 1:**
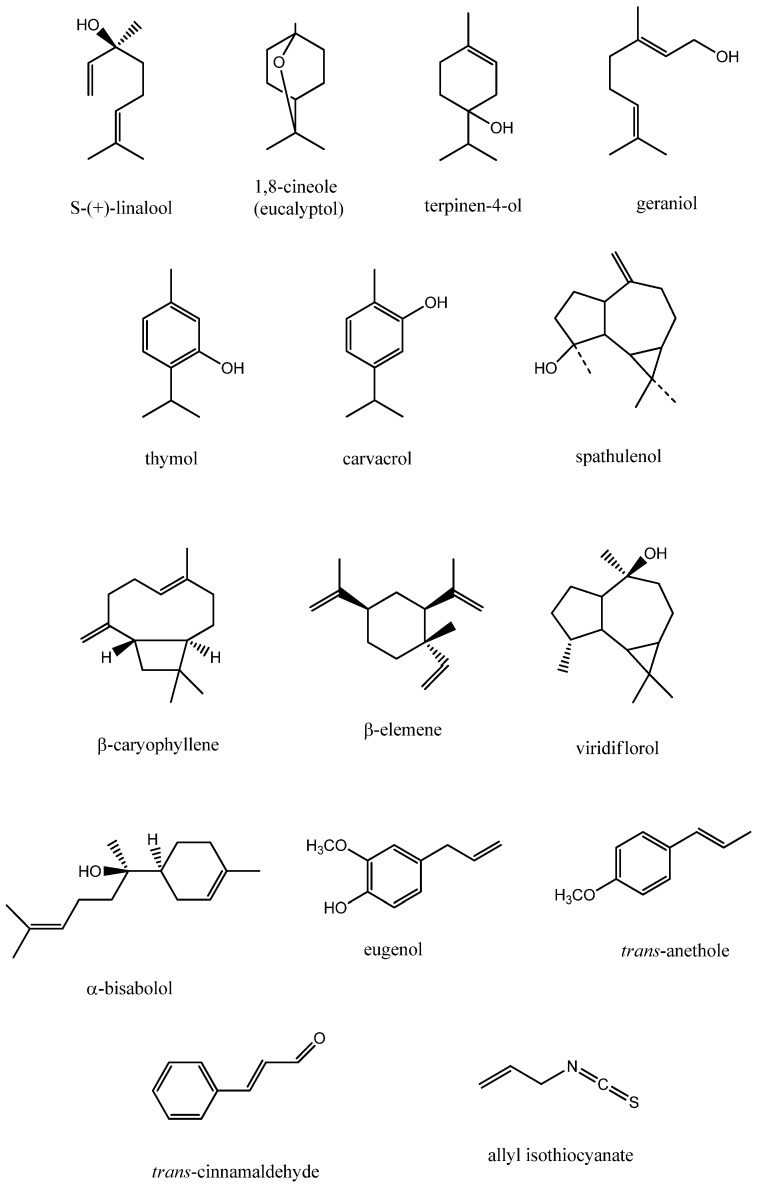
Essential oil components acting synergistically with conventional antibiotics against Gram-negative bacteria.

Both EOs and volatile compounds have been reported to modulate the antibiotic susceptibility of *A. baumannii* (Table 1). Coriander (*Coriandrum sativum* L., Apiaceae) EO acted synergistically with conventional antibiotics against two chloramphenicol-resistant *A. baumannii* reference strains (LMG 1025, LMG 1041). Coriander EO significantly increased the susceptibility to chloramphenicol of both strains as indicated by the decrease of minimum inhibitory concentration (MIC) values of chloramphenicol in combination (16- and 31-fold decrease against *A. baumannii* LMG 1025 and LMG 1041, respectively) [30]. Coriander EO has been reported to increase the permeability of bacterial membrane, thus impairing membrane potential, respiratory activity and efflux pump activity [31]. EOs are active against both Gram-positive and Gram-negative bacteria [8,32,33]. Coriander EO showed higher bactericidal effects against *A. baumannii* and other Gram-negative bacteria (*Escherichia coli*, *Klebsiella pneumoniae*, *Salmonella typhimurium*) compared to Gram-positive ones (*Bacillus cereus*, *Staphylococcus aureus*, *Enterococcus faecalis*). These differences in activity were attributed to the ability of S-(+)-linalool (Figure 1), the major constituent of coriander EO, to disrupt the negatively-charged outer membrane of Gram-negative bacteria, on the one hand, and to decrease the cell wall thickness of Gram-positive bacteria with a consequent increase in resistance to solvents and dyes, on the other hand [31]. Multidrug-resistant *A. baumannii* is an etiological agent of traumatic and burn wound infections. In these infections, hemorrhagic bullae and necrosis facilitate the entry of the pathogen into the bloodstream, leading to complications (bacteremia, septicemia). Therefore, multidrug-resistant *A. baumannii* is a major threat for patients with traumatic and burn injuries [21]. Linalool-rich EOs, such as rosewood (*Aniba rosaeodora* Ducke, Lauraceae) and myrtle (*Myrtus communis* L., Myrtaceae) EOs (60.1% and 18.32%–26.59% linalool, respectively), acted synergistically with conventional antibiotics (gentamicin, ciprofloxacin, polymyxin B) against *A. baumannii* reference strains and multidrug-resistant wound isolates [24,34]. Synergism between linalool-rich EOs and conventional antibiotics may be attributed, at least in part, to the membrane permeabilization activity of EOs, resulting in an enhanced penetration of antibiotics inside the cell [24]. Except polymyxin B, which affects the membrane structure, the other antibiotics, showing synergy in combination with linalool-rich EOs, act on different targets: chloramphenicol and gentamicin impair protein synthesis, while ciprofloxacin inhibits topoisomerase II (DNA gyrase) and IV and, consequently, DNA replication [10]. As myrtle EOs and polymyxin B target the same site, the membrane, their combination was efficient in increasing the sensitivity of multidrug-resistant *A. baumannii* isolates. Myrtle EOs and ciprofloxacin have different target sites (membrane and DNA, respectively), and therefore, their combination caused the most frequent synergistic effects [24]. Two red river gum (*Eucalyptus camaldulensis* Dehnh., Myrtaceae) EOs showed in vitro synergistic activity with polymyxin B against 22 *A. baumannii* strains (two standard strains and 20 multidrug-resistant wound isolates). Oxygenated sesquiterpene spathulenol (18.9%) and monoterpene alcohols 1,8-cineole (eucalyptol) (7.62%) and terpinen-4-ol (7.59%) (Figure 1) are mainly responsible for the red river gum EOs’ antibacterial activity. Polymyxin B-EOs combinations increased bacterial sensitivity by similar mechanisms of activity, as both act by affecting the cell membrane structures [25,35]. Both rosewood (*Aniba rosaeodora* Ducke, Lauraceae) and geranium (*Pelargonium graveolens* L., Geraniaceae) EOs (60.1% linalool and 47.3% citronellol, respectively) acted synergistically with gentamicin against *A. baumannii* ATCC 19606. The strong synergism could be favored by the two major terpenalcohols, linalool and citronellol [34]. It has been reported that such compounds are able to compromise the integrity of the cytoplasmic membrane, alter the physicochemical surface properties and determine K^+^ leakage [36]; thus, they disrupt the bacterial membrane and increase the intracellular penetration of gentamicin, a protein synthesis inhibitor [10,34,36]. Patients requiring dialysis, catheterization and mechanical ventilation, but also immunocompromised patients have a high risk of developing *A. baumannii* infections [21,22]. Immortelle (*Helichrysum italicum* (Roth) G. Don., Asteraceae) EO reduced the resistance to chloramphenicol of *A. baumannii* ATCC 19606 and the AB1 strain isolated from central venous catheters of critical care patients [37,38]. In the case of both strains, immortelle EO reduced by eight-fold the MIC value of chloramphenicol in combination, whereas phenylalanine-arginine β-naphthylamide (PaβN), a potent efflux pumps inhibitor, caused only a two-fold reduction. Geraniol (Figure 1), a component of immortelle EO, is an inhibitor of bacterial efflux pumps, which are responsible for both antibiotic resistance and resistance to host innate immune defense [37,39]. Cinnamon (*Cinnamomum zeylanicum* Nees., Lauraceae) and lemon (*Citrus limon* L. Burm. F., Rutaceae) EOs showed strong synergistic effects in combination with amikacin against a multidrug-resistant *A. baumannii* clinical isolate (A-06 strain) [40]. The study provides no information on the chemical composition of both cinnamon and lemon EOs. However, a permeabilization of the bacterial membrane by certain EO constituents followed by an increased penetration of amikacin might explain, at least in part, these synergistic effects.

## 3. Essential Oils and Volatile Compounds Modulating Antibiotic Activity against *Escherichia coli*

*Escherichia coli* is a facultative anaerobic, non-spore-forming Gram-negative bacterium, commonly abundant in the normal intestinal tract flora. Pathogenic strains are spread mainly by contaminated food and water being responsible for a wide range of infections. Identification of O (lipopolysaccharide) and H (flagellar) antigens allowed the classification of enteric *E. coli* into seven pathovars responsible for certain diseases: diarrhea in children (enteropathogenic, diffusely adherent and enteroaggregative *E. coli*), hemolytic-uremic syndrome (enterohemorrhagic *E. coli*), traveler’s diarrhea (enterotoxigenic *E. coli*), dysentery (enteroinvasive *E. coli*) and Crohn’s disease (adherent invasive *E. coli*) [41,42]. There are also two pathovars of the extraintestinal type, which are involved in urinary tract infections (uropathogenic *E. coli*) and meningitis in newborns (neonatal meningitis *E. coli*) [43]. Cell adhesion through pili or fimbriae is characteristic for all pathovars, except for enteroinvasive *E. coli.* Besides, specific virulence factors have been identified for different pathovars, such as hemolysin (uropathogenic and enteroinvasive *E. coli*) [44,45], Shiga toxins (enterohemorrhagic and enteroinvasive *E. coli*) [45] and heat-labile and heat-stable toxins (enterotoxigenic *E. coli*) [46]. *E. coli* is intrinsically resistant to penicillin G due to the outer membrane barrier, but extended spectrum β-lactamase production is responsible for the resistance to a broad spectrum of β-lactams, including penicillins, cephalosporins (third- and fourth-generation cephalosporins) and monobactams [47]. Carbapenem resistance of *E. coli* is also a therapeutic challenge because many nosocomial isolates have been reported to synthesize carbapenemases [48]. The alteration of quinolone targets (DNA gyrase, topoisomerase IV), the decrease in membrane permeability and the overexpression of efflux pumps explain the resistance of *E. coli* to quinolones [49]. The production of acetyltransferases, nucleotidyltransferases, phosphotransferases and methylation of 16S rRNA are responsible for the resistance to aminoglycosides [42]. Treatment options of infections caused by extended spectrum β-lactamase-producing *E. coli* include imipenem, meropenem and third-generation cephalosporins in combination with an enzyme inhibitor (clavulanic acid) [50]. 

A feasible approach to reduce the antibiotic resistance of *E. coli* is the combination of conventional antibiotics with EOs or volatile compounds (Table 2). A strong synergy was reported for two Moroccan thyme (*Thymus maroccanus* L. and *Thymus broussonetii* L., Lamiaceae) EOs in combination with chloramphenicol against *E. coli* strains: AG100 (wild-type *E. coli* K-12) and AG102 (AG100 derivative overexpressing AcrAB pump responsible for resistance to tetracycline, chloramphenicol, ampicillin, nalidixic acid and rifampicin) [51,52]. Both EOs decreased the MIC values of chloramphenicol against *E. coli* AG100 and AG102 strains by four- and 32-fold, respectively. The synergy could be attributed to the alteration of efflux pump activity by certain EO constituents, but also to the ability of carvacrol (76.35% in *T. maroccanus* EO, 39.77% in *T. broussonetii* EO) to cause the permeabilization and depolarization of the *E. coli* cytoplasmic membrane, thus facilitating the passage of the antibiotic to the bacterial cell [52,53]. Carvacrol (Figure 1) is a well-known antibacterial agent [54]; in the case of Gram-negative bacteria, carvacrol disintegrates the polysaccharidic capsule, followed by an increase in the fluidity and permeability of the cytoplasmic membrane [55]. In addition, it acts as a proton exchanger, thus affecting the cytoplasmic pH and proton motive force with a subsequent reduction of ATP and DNA synthesis and inhibition of cytoplasmic enzymes, leading to bacterial cell death [56,57]. Similar results were obtained for another Moroccan thyme species (*T. riatarum* L., Lamiaceae) EO in combination with chloramphenicol against *E. coli* AG100 and AG100A (AG100 derivative with AcrAB pump deleted, but resistant to kanamycin). *T. riatarum* EO decreased the MIC values of chloramphenicol against AG100 and AG100A by four- and two-fold, respectively; the synergy could be attributed to efflux pump blocking activity of *T. riatarum* EO, thus increasing the activity of chloramphenicol [58]. The lemon thyme (*Thymus* *pulegioides* L., Lamiaceae) EO showed synergistic effects with tetracycline and chloramphenicol against *E. coli* ATCC 25922. Surprisingly, the combination between lemon thyme EO and streptomycin displayed an antagonistic effect related to a possible competition for the same target: the bacterial 30S ribosomal subunit; binding of this subunit leads to inhibition of protein synthesis. Lemon thyme EO contained geraniol (66.59%) as a major constituent. Geraniol has also been reported in immortelle (*Helichrysum italicum* (Roth) G. Don, Asteraceae) EO [59]. Immortelle EO restored the chloramphenicol susceptibility of *E. coli* AG100A Tet^r^ overexpressing AcrEF efflux pumps, responsible for tetracycline resistance. The MIC value of chloramphenicol was decreased by 256-fold in combination with immortelle EO, suggesting the ability of the latter to inhibit AcrEF efflux pumps [37]. Other oxygenated monoterpene-rich EOs, such as savory (*Satureja kitaibelii* Wierzb. ex Heuff., Lamiaceae), rosewood (*Aniba rosaeodora* Ducke, Lauraceae), geranium (*Pelargonium graveolens* L., Geraniaceae) and tea tree (*Melaleuca alternifolia* (Maiden and Beach) Cheel., Myrtaceae) EOs exhibited synergistic effects in combination with different antibiotics (chloramphenicol, tetracycline, gentamicin) against *E. coli* ATCC 25922 [34,60]. Synergistic effects might be correlated with the damage of the microbial membrane lipids by terpene alcohols [34] followed by an increased penetration of antibiotics into the bacterial cell. Modulatory effects of rosemary (*Rosmarinus officinalis* L., Lamiaceae) EO on the *E. coli* EC27 clinical isolate resistant to aminoglycosides have been investigated. The antimicrobial activities of amikacin, neomycin and gentamycin were increased by rosemary EO [61]. 1,8-Cineol (eucalyptol) (Figure 1), the main component of rosemary EO, has been reported to disintegrate the outer membrane and reduce the nucleoplasm [39]. On the other hand, all three aminoglycosides bind to the 30S ribosomal subunit, blocking protein synthesis [10]. Modulation of antibiotic activity by EOs through gaseous contact is a new approach that should be taken into consideration especially, in the case of respiratory infections. Cidreira-do-mato [62], also known as cidreira-brava [63] (*Hyptis martiusii* Benth., Lamiaceae), EO increased the susceptibility to tobramycin of *E. coli* ATCC 25922 and the EC27 multidrug-resistant surgical wound isolate by 76.5% and 80%, respectively; in addition, it considerably reduced the resistance to gentamicin and amikacin of the EC27 isolate (by 40% and 82.4%, respectively). The main constituents of cidreira-brava EO were bicyclogermacrene (10.6%), *trans*-caryophyllene (9.2%), caryophyllene oxide (7.4%), 1,8-cineole (7%) and δ-2-carene (6.8%) [64]. Neither for cidreira-brava EO, nor for its main constituents studies were performed in order to assess the mechanisms of synergism with aminoglycosides, but the permeabilization of the bacterial membrane by EO components might be partially responsible for the increase in antibiotic activity. 

Several studies have reported interactions between EO constituents and conventional antibiotics against *E. coli* strains. Gallucci et al. [65] detected a potent synergism between eugenol/thymol and penicillin against *E. coli* resistant to β-lactam antibiotics. Thymol (Figure 1) also acted synergistically with penicillin against multidrug-resistant *E. coli* N00 666 [66]. These synergisms might be attributed to the ability of terpene phenols to increase the antibiotic permeation into the bacterial cell. Both thymol and carvacrol showed more synergistic interactions with antibiotics against *E. coli* strains in comparison with eugenol [66]. This difference in synergistic potential is mainly due to different electron delocalization. In the case of thymol and carvacrol, the delocalized electrons enable the release of the proton belonging to the phenolic hydroxyl group. The released proton acts as a proton exchanger and reduces the pH gradient across the cytoplasmic membrane, thus decreasing the bacterial membrane potential and causing ATP depletion [56]. Eugenol (Figure 1) has been reported to target both the bacterial membrane, leading to a non-specific increase in antibiotic penetration, and different metabolic pathways [18]. However, in the case of eugenol, the presence of the methoxyl group impairs the release of the phenolic hydroxyl proton. This structural pattern might explain not only the weaker antimicrobial activity of eugenol in comparison with thymol and carvacrol [67], but also the reduced number of synergisms between eugenol and conventional antibiotics. Cinnamaldehyde (Figure 1), a volatile phenylpropanoid, increased the antibiotic susceptibility of *E. coli* N00 666 [66]. The effect could be attributed, at least in part, to the ability of cinnamaldehyde to inhibit ATP-ase activity with a consequent alteration in bacterial membrane permeability [10,68]. An antagonistic interaction against *E. coli* has been reported for myrcene-penicillin combination [65]. Myrcene is a monoterpene present in different percentages in EOs isolated from hemp (*Cannabis sativa* L., Cannabaceae) (21%–35%) [69], hop (*Humulus lupulus* L., Cannabaceae) (6%–10%) [70], chameleon plant/Chinese lizard tail/fishwort/heartleaf (*Houttuynia cordata* Thunb., Saururaceae) (up to 30.8%) [71,72] and lemon grass (*Cymbopogon citratus* (DC.) Stapf., Poaceae) (3%–8%) [73]. The antagonism might be attributed to a competition between myrcene and penicillin for the same targets, namely penicillin-binding proteins [10]. 

## 4. Essential Oils and Volatile Compounds Modulating Antibiotic Activity against *Klebsiella pneumoniae*

*Klebsiella pneumoniae* is a rod-shaped, encapsulated, non-motile, facultative anaerobic and lactose-fermenting bacillus, an opportunistic pathogen, being asymptomatically present in the gastrointestinal tract, mouth, nasopharynx and skin of healthy persons [77,78]. This Gram-negative bacterium is associated with both community-acquired and nosocomial infections, such as severe forms of pneumonia, occurring mainly in alcoholic and diabetic patients [78], pyogenic liver abscess with severe extrahepatic metastasis, including septic endophthalmitis and meningitis [79], urinary tract infections [80], nosocomial pneumonia, wound and surgical site infections, peritonitis and endocarditis, followed by subsequent bacteremia [78,81]. *K. pneumoniae* virulence factors include the capsule polysaccharides, fimbriae, outer membrane lipopolysaccharide and proteins and siderophores [78]. The capsule polysaccharides express antiphagocytic functions and resistance to the lytic activity of the serum complement, being essential for bacterial pathogenicity [82]. The severity of *K. pneumoniae* infections is also due to the biofilm formation on both biotic and abiotic surfaces [78,82,83]. Over the last two decades, numerous reports have described *K. pneumoniae* carbapenemase-producing strains. The production of these extended-spectrum β-lactamases and modification of the outer membrane permeability (porin defects, upregulation of efflux pumps) are responsible for the emergence of multidrug-resistant *K. pneumoniae* phenotypes [84,85]. As carbapenemases are able to hydrolyze almost all β-lactam antibiotics and even β-lactamase inhibitors, carbapenem-resistant *K. pneumoniae* remains susceptible only to a few second-line antibiotics, such as colistin, tigecycline, fosfomycin, polymyxin B and gentamicin, administered alone or in combination [83,86]. Recently, colistin-resistant *K. pneumoniae* has been reported in Italian hospitals [87]. In the case of infections caused by carbapenem-resistant *K. pneumoniae*, the combination therapy including a carbapenem and a second-line antibiotic significantly improved the patient survival [85,88]. Selection of second-line anti-*K. pneumoniae* agents should also consider particular toxicity risks for patients (e.g., colistin is known for its nephrotoxic and neurotoxic effects, and gentamicin possesses the risk of nephrotoxicity) [85].

Several EOs and volatile compounds have been reported to increase the susceptibility of *K. pneumoniae* reference strains or clinical isolates to conventional antibiotics (Table 3). A study investigating two endemic Moroccan thyme (*Thymus maroccanus* L. and *Thymus broussonetii* L., Lamiaceae) EOs reported synergistic effects in combination with conventional antibiotics against a *K. pneumoniae* clinical isolate. *T. maroccanus* EO acted synergistically with ciprofloxacin, gentamicin and pristinamycin, while *T. broussonetii* EO acted synergistically only with pristinamycin. The different behavior of these two EOs may be attributed to their different chemical composition: *T. maroccanus* EO contained a higher amount of carvacrol (76.35%) than *T. broussonetii* EO (39.77%) [57]. *Thymus saturejoides* Coss. (Lamiaceae) EOs, containing carvacrol as the main constituent (25.3%–45.3%), acted synergistically with cefixime against a *K. pneumoniae* clinical isolate [76]. These synergistic effects are due to multiple targets that the combinations act on, such as the bacterial membrane (carvacrol, cefixime) [10,56], proteins (gentamicin, pristinamycin) [10], enzymes (carvacrol) [89], ATP (carvacrol) [55,56] and DNA (carvacrol, ciprofloxacin) [10,56]. Combinations between savory (*Satureja kitaibelii* Wierzb. ex Heuff., Lamiaceae) EO and two conventional antibiotics (chloramphenicol and tetracycline) were found to be synergistic against *K. pneumoniae* ATCC 700603 (10-fold reduction in the MIC values of both antibiotics). However, geraniol, the main component of savory EO (50.4%), showed synergistic effects only in combination with chloramphenicol (10-fold reduction in the MIC value), exhibiting similar antibacterial activity to that of savory EO. As mentioned before, geraniol is a bacterial resistance modulator by targeting efflux pumps involved in antibiotic resistance [37,60]. Additive effects were recorded for geraniol-tetracycline combinations; it seems that other compounds in savory EO (limonene, ocimene, linalool, nerol, β-caryophyllene, germacrene D) might be involved in the synergistic interaction with tetracycline [60]. Lemon thyme (*Thymus pulegioides* L., Lamiaceae) EO, belonging to geraniol/geranyl acetate chemotype, showed synergistic effects with tetracycline, streptomycin and chloramphenicol against the same reference strain, *K. pneumoniae* ATCC 700603 [74]. Miladinović et al. [90] reported synergistic effects between moon carrot (*Libanotis montana* Crantz, Apiaceae) EO and chloramphenicol against *K. pneumoniae* ATCC 700603 (10-fold reduction in the MIC value of chloramphenicol). The main components of moon carrot EO were sesquiterpene hydrocarbons (67.2%), β-elemene (Figure 1) being the major constituent (40.4%) [90]. Due to its structural analogy with cyclohexane, β-elemene disrupts the outer membrane of Gram-negative bacteria and alters the permeability of cytoplasmic and mitochondrial membranes [91]. Thus, β-elemene acts as a membrane permeabilizer and favors the chloramphenicol intrinsic mechanism of activity, namely the inhibition of protein synthesis [10,92].

## 5. Essential Oils and Volatile Compounds Modulating Antibiotic Activity against *Pseudomonas aeruginosa*

*Pseudomonas aeruginosa* is an aerobic, rod-shaped, non-spore forming, oxidase positive and non-lactose fermenting Gram-negative bacterium [93]. It is a common agent for nosocomial infections being responsible for urinary tract, corneal, surgical and wound infections, but also for pneumonia and endocarditis. This pathogen is involved in chronic respiratory infections in cystic fibrosis patients. In addition, it is the second pathogen responsible for ventilator-associated pneumonia [94]. Bacteremia caused by *P. aeruginosa* leads to a mortality rate of 30%–50% in cancer patients who develop neutropenia after chemotherapy [95,96]. The pili, proteases, exoenzyme S, siderophores and alginate pseudocapsule are the main virulence factors of *P. aeruginosa* [97]. Amoxicillin, first and second generation cephalosporins are naturally inactive against *P. aeruginosa.* The most problematic aspect is the resistance of *P. aeruginosa* strains to many antibiotics. Piperacillin and ceftazidime resistance is explained by β-lactamase production, but strains resistant to quinolones and aminoglycosides have also been reported [95]. Besides the alteration of antibiotic target, another mechanism responsible for antibiotic resistance is the efflux pump overexpression [98].

Numerous studies have been conducted in order to assess if combinations between EOs and conventional antibiotics are able to increase the antibiotic susceptibility of *P. aeruginosa* strains (Table 4). Strong synergistic effects have been reported for the combinations between basil (*Ocimum basilicum* L., Lamiaceae) EO and imipenem/ciprofloxacin against *P. aeruginosa* ATCC 25853 and the 1662339 clinical isolate. Both linalool, the main ingredient of basil EO (55.2%), and imipenem act on the same target, the bacterial cell membrane, whereas ciprofloxacin inhibits DNA gyrase and topoisomerase IV [99,100]. Thyme (*Thymus vulgaris* L., Lamiaceae) and marjoram (*Origanum majorana* L., Lamiaceae) EOs restored piperacillin susceptibility of *P. aeruginosa* ATCC 9027 (resistant to piperacillin, but susceptible to cefepime, meropenem, gentamicin and norfloxacin). Both EOs acted synergistically in combination with cefepime, meropenem and gentamicin. Thyme and marjoram EOs had high contents of oxygenated terpenes, thymol (33.6%) and 4-terpineol (21.3%), respectively. Weaker synergistic effects have been detected for sage (*Salvia officinalis* L., Lamiaceae) EO in combination with piperacillin, meropenem and gentamicin. 1,8-Cineole (29%) (Figure 1) was the major oxygenated terpene in sage EO [101]. Alecrim pimento (*Lippia sidoides* Cham*.*, Verbenaceae) EO, rich in thymol (84.9%), reduced the MIC values of gentamicin and neomycin by four- and two-fold, respectively [102]. Phenolic terpenoids, such as thymol, alter the bacterial membrane, facilitating the antibiotic uptake. Moreover, thymol is distributed in the bilayer lipid membrane causing loss of K^+^ and ATP [103]. Additional mechanisms involve the alteration of periplasmic proteins and the citrate metabolic pathway [104]. Monoterpene alcohols, such as 4-terpineol, also have antipseudomonal activity, as they inhibit the microbial oxygen uptake and oxidative phosphorylation. Terpenoid ethers (1,8-cineole) have a lower capacity to sensitize the pathogen to the antibiotic activity as they lack free hydroxyl groups, a structural feature present in both phenolic and alcoholic terpenoids [101,105]. Three *Thymus saturejoides* Coss. (Lamiaceae) EOs significantly increased the susceptibility of AmpC β-lactamase producing *P. aeruginosa* ATCC 27853 to cefixime. All EOs were characterized by high contents of carvacrol (25.3%, 26.5% and 45.3%) and borneol (19.7%, 20.1% and 7.5%) [76]. Other carvacrol-rich EOs, *Thymus maroccanus* L. and *Thymus broussonetii* L. (Lamiaceae) EOs, acted synergistically with ciprofloxacin and gentamicin against a *P. aeruginosa* clinical isolate. These synergistic effects are mainly due to carvacrol, a cytoplasmic membrane permeabilizer and an ATP, DNA and enzyme inhibitor [55,56,57]. *Lippia gracilis* Schauer. (Verbenaceae) EO, containing thymol (44.4%) and carvacrol (22.2%) as major constituents, significantly increased the susceptibility of *P. aeruginosa* ATCC 15442 to amikacin, tobramycin and gentamicin if the combinations were tested by direct contact. A marked difference was detected when tested in gaseous contact: high dilutions of *Lippia gracilis* EO (25%, 12% and 6%) increased the activity of gentamycin, but did not increase the activity of tobramycin. Tobramycin and gentamicin belong to the same class of antibiotics, but tobramycin is more polar than gentamicin, and it can hardly cross the lipid membrane. Moreover, in contrast with direct contact, in the case of gaseous contact, the interaction between bacteria and EO vapors is significantly influenced by the concentration of the latter [106]. It is possible that the high dilutions of *Lippia gracilis* EO used in the study were not enough to facilitate the entrance of tobramycin within the cell [107]. The susceptibility of *P. aeruginosa* ATCC 15442 to tobramycin, amikacin and gentamicin was increased by gaseous contact with *Hyptis martiusii* Benth. (Verbenaceae) EO. The synergistic effect with amikacin in gaseous contact was converted into an antagonistic one in the case of direct contact. A possible explanation would be related to the fact that the gaseous contact involves a series of qualitative and quantitative changes in EO composition. Unstable volatile constituents undergo degradation, generating new substances with different biological effects; for example, bicyclogermacrene converts into spathulenol, which increases the antibacterial activity of *Hyptis martiusii* EO [64]. Synergistic effects against the same β-lactamase producing strain (*P. aeruginosa* ATCC 27853) have been reported for geraniol, savory (*Satureja kitaibelii* Wierzb. ex Heuff., Lamiaceae) and lemon thyme (*Thymus pulegioides* L., Lamiaceae) EOs in combination with chloramphenicol or tetracycline. Geraniol is a major constituent in savory and lemon thyme EOs (50.4% and 66.59%, respectively) [60,74]. Immortelle (*Helichrysum italicum* (Roth) G. Don., Asteraceae) EO restored the susceptibility to chloramphenicol of two highly chloramphenicol-resistant *P. aeruginosa* strains (PAO1 and PA124). Immortelle EO decreased the MICs of chloramphenicol against PAO1 and PA124 by 16- and eight-fold, respectively. These effects are undoubtedly due to the ability of geraniol, a constituent of EO, to inhibit efflux pumps responsible for drug resistance. As a result, geraniol increased the intra-bacterial concentration of antibiotics [37]. Nevertheless, lemon thyme EO acted antagonistically with streptomycin. Antagonism is often due to a competition for the same target. It is possible that some constituents in lemon thyme EO have the same target as streptomycin, namely the 30S ribosomal subunit [74]. *P. aeruginosa* ATCC 15442 is usually used to assess the effectiveness of disinfectants and antimicrobial preservatives. Gentamicin activity against this bacterial strain was significantly enhanced by gaseous contact with oxygenated sesquiterpene-rich EOs (*Zanthoxylum articulatum*, *Vanillosmopsis arborea*, *Croton zehntneri* EOs) [106,108,109]. Oxygenated sesquiterpenes (viridiflorol, α-bisabolol, *trans*-anethole) (Figure 1) are lipophilic compounds that accumulate into the phospholipid bilayers of bacterial membrane, causing its disruption, with a subsequent increase in the penetration of hydrophilic substances, such as gentamicin [110,111]. Viridiflorol exerts antibacterial activity through an additional mechanism, including interference with the respiratory chain reaction and energy production [106]. Antagonistic effects have been detected for *Vanillosmopsis arborea* EO in combination with tetracycline or tobramycin (gaseous contact). These effects could be attributed to chemical interactions between EO constituents and tetracycline/tobramycin, which led to antibiotic inactivation [108]. Similar results have been reported for *Lippia microphylla* EO, rich in 1,8-cineole (18.1%) and β-ocimene (15.2%) [112]. A strong synergistic effect against *P. aeruginosa* ATCC 15442 was recorded for amikacin in association with *Lantana montevidensis* Briq. (Verbenaceae) EO containing 31.5% β-caryophyllene (Figure 1). This non-polar compound might also affect the bacterial membrane and interfere with the mitochondrial respiratory chain reaction [113,114]. It is worthy to note eugenol (Figure 1), a volatile phenylpropanoid, which enhanced the activity of different penicillins, fluoroquinolones and macrolides against *P. aeruginosa* NCIM 5029. Bacterial cells treated with eugenol at a concentration of 1 mM had a 50% loss of membrane integrity. Therefore, eugenol synergized with penicillins, antibiotics targeting the bacterial wall. Acting as a membrane permeabilizer, eugenol facilitated the uptake of antibiotics that target ribosome (erythromycin, tetracycline, chloramphenicol, rifampicin) or DNA synthesis (norfloxacin). The synergistic effect could also be attributed to the intrinsic antimicrobial activity of eugenol. The hydroxyl group of eugenol is responsible for the inhibition of some enzymes, such as ATP-ase, histidine decarboxylase or proteases, thus impairing the bacterial metabolism [104,115].

## 6. Essential Oils and Volatile Compounds Modulating Antibiotic Activity against Other Gram-Negative Bacteria

*Enterobacter aerogenes* and *E. cloacae* are facultative anaerobic, rod-shaped and non-spore-forming Gram-negative bacteria with clinical significance, being responsible for hospital-acquired infections [116,117]. These infections are usually associated with a high fatality rate due to antibiotic resistance. *E. aerogenes* and *E. cloacae* are naturally resistant to aminopenicillins, and numerous clinical isolates exhibited resistance to other β-lactam antibiotics due to the production of extended-spectrum β-lactamases. Analysis of several *E. aerogenes* clinical isolates revealed resistance to quinolones, tetracycline and chloramphenicol due to the overexpression of efflux mechanisms [118]. Several EOs have been evaluated in order to assess their potential as antibiotic resistance modifying agents for *E. aerogenes* and *E. cloacae* strains (Table 5). *Thymus maroccanus* L. and *Thymus broussonetii* L. (Lamiaceae) EOs decreased the MIC value of chloramphenicol against *E. aerogenes* ATCC 13048 and the EA27 clinical isolate (overexpressing AcrAB pump responsible for multidrug-resistance) by four- and eight-fold, respectively. The results were comparable to those obtained with PaβN. It seems that both EOs inhibited the efflux pumps [52]. For *T. maroccanus* EO, there has also been reported a membranotropic effect on Gram-negative bacteria involving the permeabilization of both inner and outer bacterial membranes, whereas polymyxin B permeabilizes only the outer membrane; no degradation of cellular constituents was detected [119]. Both thyme EOs acted synergistically with ciprofloxacin, gentamicin and pristinamycin against an *E. cloacae* clinical isolate [57]. Carvacrol, the main component of both EOs (76.35% and 39.77%, respectively), is known to increase the bacterial membrane permeability and inhibit ATP-ase activity [10], thus facilitating the activity of conventional antibiotics that act on different targets. Immortelle (*Helichrysum italicum* (Roth) G. Don., Asteraceae) EO increased the susceptibility to chloramphenicol of several *E. aerogenes* strains: *E. aerogenes* ATCC 13048 (susceptible to many antibiotics, but resistant to amoxicillin), *E. aerogenes* EAEP289 (overexpressing the AcrAB pump, but susceptible to kanamycin), *E. aerogenes* EAEP294 (having the AcrAB pump deleted, but expressing other efflux pumps involved in antibiotic resistance) and *E. aerogenes* CM-64 (overexpressing the AcrAB pump). It is worthy to note a significant reduction in the MIC of chloramphenicol against the *E. aerogenes* EAEP294 strain (128-fold reduction). Geraniol, a component of immortelle EO, has been reported to increase the susceptibility of *E. aerogenes* EAEP294 to other antibiotics (ampicillin, penicillin, norfloxacin) [37]. Obviously, the efflux pump inhibitory activity of geraniol plays an important role in the synergy between immortelle EO and antibiotics against *E. aerogenes* strains. Synergistic effects against *E. aerogenes* NCIM 5139 have been reported for eugenol in combination with antibiotics acting on different targets [115,120].

*Proteus vulgaris* is an aerobic and facultatively anaerobic member of the *Enterobacteriaceae* family. Urinary tract, burn, bloodstream, wound and respiratory tract infections are the most common infections caused by *P. vulgaris.* Adherence through pili or fimbriae and cytotoxic hemolysins are the most important pathogenicity factors of *P. vulgaris* [121,122]. *P. vulgaris* is naturally resistant to benzylpenicillin, oxacillin, macrolides and tetracycline. Acquired resistance has been reported for ampicillin, third-generation cephalosporins (ceftazidime, cefotaxime, ceftriaxone), but also for aztreonam, monobactam due to extended-spectrum β-lactamase production. *P. vulgaris* strains are usually susceptible to aminoglycosides and quinolones [123]. Eugenol has been reported to act synergistically in combination with several antibiotics against *P. vulgaris* NCIM 2813 (Table 5).

*Salmonella* spp. are motile Gram-negative bacteria. Ninety-nine-point-five percent of the pathogenic *Salmonella* strains belong to *S. enterica* subsp. *enterica*. Infections caused by *Salmonella* are treated with ampicillin, chloramphenicol and trimethoprim-sulfamethoxazole, but multidrug-resistant strains have also been isolated. Fluoroquinolones, extended-spectrum cephalosporins [125] and azithromycin [126] have been used in order to treat multidrug-resistant *S. enterica* subsp. *enterica* serovar *typhi* (*S. typhi*). Synergistic effects between EOs or volatile compounds and antibiotics against *Salmonella* strains have been reported (Table 5). The combinations between *Thymus maroccanus* EO and ciprofloxacin or cefixime, *Thymus broussonetii* EO and pristinamycin or cefixime exhibited total synergism against *Salmonella* sp. CCMM B_17_ (laboratory collection) [57]. As carvacrol, the main component of both EOs, and nalidixic acid (at low concentrations) target the bacterial membrane; their combination significantly increased the susceptibility of nalidixic acid-resistant *Salmonella* strains (including *S. typhimurium*, *S. derby*, *S. enteritidis*, *S. minnesota)* [124]. Both carvacrol and thymol acted synergistically with different antibiotics (ampicillin, penicillin, tetracycline, erythromycin, bacitracin, novobiocin) against tetracycline-resistant *S. typhimurium* SGI1, whereas eugenol acted synergistically only with tetracycline and novobiocin [66]. In a study conducted by Hemaiswarya et al. [115], eugenol was found to increase the antibiotic susceptibility of *S. typhimurium* NCIM 2501. Allyl isothiocyanate (Figure 1), a constituent of EOs isolated from Brassicaceae species [127], acted synergistically in combination with ampicillin, erythromycin and bacitracin against *S typhimurium* SGI1 [66]. Allyl isothiocyanate targets the same bacterial element as ampicillin and bacitracin: the cell wall [128]. Besides, the central highly electrophile carbon atom of allyl isothiocyanate is a structural pattern that induces the damage of cytoplasmic proteins [104]. Proteic damage caused by allyl isothiocyanate could explain the synergism with erythromycin (inhibitor of protein synthesis through 50S ribosomal subunit binding) [10].

## 7. Conclusions 

In conclusion, EOs and their components can enhance the antibiotic activity against Gram-negative bacteria. Most volatile compounds disrupt the bacterial membrane, thus facilitating antibiotic penetration. Enzyme inhibition, reduction of ATP and DNA synthesis and alteration of metabolic pathways are other mechanisms by which EOs and pure volatile compounds act in synergy with conventional antibiotics against Gram-negative bacteria. By inhibiting efflux pumps, volatile compounds, such as geraniol, restore the antibiotic susceptibility of Gram-negative-resistant strains. The mechanisms of synergy of EOs with conventional antibiotics are very complex and still not completely understood as they involve multiple interactions between individual EO constituents, on the one hand, and individual EO constituents and antibiotics, on the other hand. EOs usually possess different antibacterial effects than their major components, which suggests synergistic, additive or antagonistic interactions between their constituents and also an undoubted contribution of minor components to the antibacterial activity. For example, eucalyptus and lavender EOs showed higher antibacterial effects against *Staphylococcus aureus*, *Bacillus subtilis* and *Escherichia coli* compared to their major components, 1,8-cineole and linalyl acetate, respectively. On the contrary, thyme EO was less active against the same bacterial strains than its main constituent, thymol [129]. Both thymol and carvacrol have been detected in EOs isolated from certain *Lippia*, *Thymus* and *Origanum* species [57,76,107,130,131,132,133]. Surprisingly, these two monoterpene phenols might modulate the antibiotic activity in a different manner. For instance, thymol showed excellent synergistic effects with penicillin against *Escherichia coli* (resistant to some β-lactam antibiotics), whereas the combination of carvacrol with penicillin resulted in an indifferent effect [65]. The complexity of interactions generated when combining EOs and antibiotics is evident. Overall, the results of the in vitro assays are very promising for the development of new effective formulations against Gram-negative bacteria. Obviously, further in vivo studies are required to evaluate the bioavailability, efficacy and toxicity of EO/volatile compound-antibiotic combinations.

## Figures and Tables

**Table 1:** Essential oils (EOs) and volatile compounds modulating the antibiotic activity against *Acinetobacter baumannii*.

Essential Oil/Volatile Compound	Vegetal Source	Major Constituent	*Acinetobacter baumannii* Strain	Antibiotic	Method	Effect	Ref.
Tea tree EO	*Melaleucae folium* (*Melaleuca alternifolia* (Maiden. and Beach.) Cheel., Myrtaceae, Tea tree)	Terpinen-4-ol (30.3%)	ATCC 19606	Gentamicin	CB (FICI = 0.5)	S	[34]
Myrtle EO	*Myrti folium* (*Myrtus communis* L., Myrtaceae, Myrtle)	Linalool (22.276%) Myrtenyl acetate (16.561%) 1,8-Cineole (13.250%)	ATCC 19606 ATCC BAA747 and 22 clinical isolates of which MDR strains: Aba-6637, Aba-4914, Aba-5055	Ciprofloxacin	CB (FICI < 0.5) TK	S	[24]
		Linalool (18.320%) Myrtenyl acetate (18.009%) 1,8-Cineole (16.878%)					
				Polymyxin B		S	
		Linalool (26.591%) Myrtenyl acetate (18.489%) 1,8-Cineole (15.762%)					
Longbeak eucalyptus EO	*Eucalypti folium* (*Eucalyptus camaldulensis* Dehnh., Myrtaceae, Longbeak eucalyptus)	Spathulenol (18.90%)	Aba-4914 Aba-5055	Ciprofloxacin	CB (FICI < 0.5)	S	[25]
			Aba-6673		CB (FICI = 0.53)	Ad	
			Aba-4914 Aba-6673	Gentamicin	CB (FICI < 0.5)	S	
		Spathulenol (21.39%)					
					CB (FICI = 0.5)	Ad	
			ATCC 19606 Aba-4914 Aba-5055	Polymyxin B	CB (FICI < 0.5) TK	S	
Rosewood EO	*Anibae* *lignum* (*Aniba rosaeodora* Ducke, Lauraceae, Rosewood)	Linalool (60.1%)	ATCC 19606	Gentamicin	CB (FICI = 0.11)	S	[34]
Cinnamon EO	*Cinnamomi cortex* (*Cinnamomum zeylanicum* Nees, Lauraceae, Cinnamon)	-	Clinical isolate A-06	Amikacin	CB (FICI = 0.045)	S	[40]
				Gentamicin	CB (FICI = 0.5)	Ad	
				Imipenem	CB (FICI = 2)	I	
				Meropenem	CB (FICI = 1.5)	I	
Coriander EO	*Coriandri fructus* (*Coriandrum sativum* L., Apiaceae, Coriander)	-	LMG 1025	Cefoperazone	CB (FICI = 0.750)	Ad	[30]
				Chloramphenicol	CB (FICI = 0.312)	S	
				Ciprofloxacin	CB (FICI = 0.281)	S	
				Gentamicin	CB (FICI = 0.250)	S	
				Piperacillin	CB (FICI = 1)	Ad	
				Tetracycline	CB (FICI = 0.312)	S	
			LMG 1041	Cefoperazone	CB (FICI = 1)	Ad	
				Chloramphenicol	CB (FICI = 0.047)	S	
				Ciprofloxacin	CB (FICI = 0.375)	S	
				Gentamicin	CB (FICI = 0.375)	S	
				Piperacillin	CB (FICI = 0.625)	Ad	
				Tetracycline	CB (FICI = 0.185)	S	
Immortelle EO	*Helichrysi flos* (*Helichrysum italicum* (Roth) G. Don, Asteraceae, Immortelle)	-	ATCC 19606 Clinical isolate AB1	Chloramphenicol	FR (8-fold reduction in MIC)	S	[37]
Oregano EO	*Origani herba* (*Origanum vulgare* Linn., Lamiaceae, Oregano)	Cymenol (58.6%)	ATCC 19606	Gentamicin	CB (FICI = 0.65)	Ad	[34]
Geranium EO	*Pelargonii herba* (*Pelargonium graveolens* L., Geraniaceae, Geranium)	Citronellol (47.3%)	ATCC 19606	Gentamicin	CB (FICI = 0.11)	S	
Lemon EO	*Citri pericarpium* (*Citrus limon* L. Burm. F., Rutaceae, Lemon)	-	Clinical isolate A-06	Amikacin	CB (FICI = 0.037)	S	[40]
				Gentamicin	CB (FICI = 0.5)	Ad	
				Imipenem	CB (FICI = 2)	I	
				Meropenem	CB (FICI = 2)	I	

Ad: addition; S: synergy; I: indifference; CB: checkerboard assay; TK: time kill assay; FICI: fractional inhibitory concentration index; FR: fold reduction in MIC; MIC: minimum inhibitory concentration; MDR: multidrug-resistant.

**Table 2:** Essential oils and volatile compounds modulating the antibiotic activity against *Escherichia coli*.

Essential Oil/Volatile Compound	Vegetal Source	Major Constituent	*Escherichia coli* Strain	Antibiotic	Method	Effect	Ref.
Oregano EO	*Origani herba* (*Origanum vulgare* Linn., Lamiaceae, Oregano)	Cymenol (58.6%)	ATCC 25922	Gentamicin	CB (FICI = 0.65)	Ad	[34]
Thyme EO	*Thymi herba* (*Thymus maroccanus* L., Lamiaceae, Za’ater/Azukeni)	Carvacrol (76.35%)	AG100	Chloramphenicol	FR (4-fold reduction in MIC)	S	[52]
			AG102		FR (32-fold reduction in MIC)		
	*Thymi herba* (*Thymus broussonetii* L., Lamiaceae, Za’atar Essaouiri)	Carvacrol (39.77%) Borneol (12.03%)	AG100		FR (4-fold reduction in MIC)		
			AG102		FR (32-fold reduction in MIC)		
Lemon thyme EO	*Thymi herba* (*Thymus pulegioides* L., Lamiaceae, Lemon thyme/broad-leaved thyme)	Geraniol (66.59%)	ATCC 25922	Tetracycline	CB (FICI = 0.43–0.76)	S	[74]
					CB (FICI = 0.98–1.09)	Ad	
				Streptomycin	CB (FICI = 1.20–2.80)	A	
				Chloramphenicol	CB (FICI = 0.21–0.87)	S	
					CB (FICI = 0.98–1.09)	Ad	
Savory EO	*Saturejae herba* (*Satureja kitaibelii* Wierzb. ex Heuff., Lamiaceae, Savory)	Geraniol (50.4%)	ATCC 25922	Chloramphenicol	CB (FICI = 0.21–0.87)	S	[60]
					CB (FICI = 0.98–1.09)	Ad	
				Tetracycline	CB (FICI = 0.32–0.87)	S	
					CB (FICI = 0.98–1.09)	Ad	
Sandarac EO	*Tetraclinis coni* (*Tetraclinis articulata* (Vahl.) Masters., Cupressaceae, Sandarac)	α-Campholenal (16.34%) *Trans*-Pinocarveol (15.45%) Verbenone (13.36%) *Cis*-Verbenol (12.36%)	ATCC 10536	Amoxicillin	CB (FICI = 1)	Ad	[75]
Rosemary EO	*Rosmarini folium* (*Rosmarinus officinalis* L., Lamiaceae, Rosemary)	1,8-Cineole (30.87%)	Clinical isolate	Amikacin	FR (~250-fold reduction in MIC)	S	[61]
				Neomycin	FR (~250-fold reduction in MIC)		
				Gentamicin	FR (~4200-fold reduction in MIC)		
Cidreira-do-mato/cidreira-brava EO	*Hyptis folium* (*Hyptis martiusii* Benth., Lamiaceae, Cidreira-do-mato/cidreira-brava)	Bicyclogermacrene (10.6%) *Trans*-Caryophyllene (9.2%) Caryophyllene oxide (7.4%) 1,8-Cineole (7%)	ATCC 25922	Amikacin	FR (–)	I	[64]
					IZGC (+2.4%)	S	
				Gentamycin	FR (–)	I	
					IZGC (+17.6%)	S	
				Neomycin	FR (4-fold reduction in MIC)	S	
				Kanamycin	FR (–)	I	
				Tobramycin	IZGC (+76.5%)	S	
Cidreira-do-mato/cidreira-brava EO	*Hyptis folium* (*Hyptis martiusii* Benth., Lamiaceae, Cidreira-do-mato/cidreira-brava)	Bicyclogermacrene (10.6%) *Trans*-Caryophyllene (9.2%) Caryophyllene oxide (7.4%) 1,8-Cineole (7%)	Clinical isolate	Amikacin	FR (–)	I	[64]
					IZGC (+82.4%)	S	
				Gentamycin	FR (–)	I	
					IZGC (+40.0%)	S	
				Neomycin	FR (–)	I	
				Kanamycin	FR (–)	I	
				Tobramycin	IZGC (+80.0%)	S	
Thyme EO	*Thymi herba* (*Thymus riatarum* Humbert and Maire., Lamiaceae, Moroccan thyme)	Borneol (41.67%)	AG100	Chloramphenicol	FR (4-fold reduction in MIC)	S	[58]
			AG100A		FR (2-fold reduction in MIC)		
Thyme EO	*Thymi herba* (*Thymus saturejoides* Coss., Lamiaceae, Azoukni)	Carvacrol (25.3%) Borneol (19.7%)	ATCC 25922	Cefixime	CB (FICI = 1.25)	I	[76]
		Carvacrol (26.5%) Borneol (20.1%)					
		Carvacrol (45.3%) Borneol (7.5%)					
Rosewood EO	*Anibae* *lignum* (*Aniba rosaeodora* Ducke, Lauraceae, Rosewood)	Linalool (60.1%)	ATCC 25922	Gentamicin	CB (FICI = 0.35)	S	[34]
Geranium EO	*Pelargonii herba* (*Pelargonium graveolens* L’Hér., Geraniaceae, Geranium)	Citronellol (47.3%)	ATCC 25922	Gentamicin	CB (FICI = 0.30)	S	[34]
Tea tree EO	*Melaleucae folium* (*Melaleuca alternifolia* (Maiden. and Beach.) Cheel., Myrtaceae, Tea tree)	Terpinen-4-ol (30.3%)	ATCC 25922	Gentamicin	CB (FICI = 0.49)	S	
Citronellol	-		Clinical isolate	Penicillin	CB (FICI = 1.03)	I	[65]
Geraniol	-		Clinical isolate	Penicillin	CB (FICI = 1.5)	I	[60]
			ATCC 25922	Chloramphenicol	CB (FICI = 0.32–0.87)	S	
					CB (FICI = 0.98–1.09)	Ad	
				Tetracycline	CB (FICI = 0.32–0.87)	S	
					CB (FICI = 0.98–1.28)	Ad	
Menthol	-		Clinical isolate	Penicillin	CB (FICI = 1.5)	I	[65]
Myrcene	-		Clinical isolate	Penicillin	CB (FICI = 10)	A	
Thymol	-		Clinical isolate	Penicillin	CB (FICI = 0.15)	S	[65]
			N00 666	Ampicillin	CB (FICI = 0.12)	S	[66]
				Penicillin	CB (FICI = 0.20)		
				Tetracycline	CB (FICI = 0.15)		
				Erythromycin	CB (FICI = 0.25)		
				Bacitracin	CB (FICI = 0.56)	I	
				Novobiocin	CB (FICI = 0.37)	S	
Eugenol	-		Clinical isolate	Penicillin	CB (FICI = 0.16)	S	[65]
			N00 666	Ampicillin	CB (FICI > 0.5)	I	[66]
				Penicillin			
				Tetracycline	CB (FICI = 0.16)	S	
				Erythromycin	CB (FICI = 1.1)	I	
				Bacitracin	CB (FICI = 0.5)	S	
				Novobiocin	CB (FICI = 1.1)	I	
Carvacrol	-		Clinical isolate	Penicillin	CB (FICI = 2)	I	[65]
			N00 666	Ampicillin	CB (FICI = 0.25)	S	[66]
				Penicillin	CB (FICI = 0.37)		
				Tetracycline	CB (FICI = 0.15)		
				Erythromycin	CB (FICI = 1.0)	I	
				Bacitracin	CB (FICI = 0.25)	S	
				Novobiocin	CB (FICI = 0.63)	I	
Cinnamaldehyde	-		N00 666	Ampicillin	CB (FICI = 0.37)	S	[66]
				Penicillin	CB (FICI = 0.24)		
				Tetracycline	CB (FICI = 0.37)		
				Erythromycin	CB (FICI = 0.24)		
				Bacitracin	CB (FICI = 0.63)	I	
				Novobiocin	CB (FICI = 0.24)	S	
Allyl isothiocyanate	-		N00 666	Ampicillin	CB (FICI = 0.63)	I	
				Penicillin	CB (FICI = 1.0)		
				Tetracycline	CB (FICI = 0.75)		
				Erythromycin	CB (FICI = 0.73)		
				Bacitracin	CB (FICI = 0.5)	S	
				Novobiocin	CB (FICI = 1.0)	I	

A: antagonism; Ad: addition; I: indifference; S: synergy; CB: checkerboard assay; FICI: fractional inhibitory concentration index; FR: fold reduction in MIC; MIC: minimum inhibitory concentration; IZGC (%): percentage change in inhibition zone diameter due to gaseous contact with essential oil/volatile component.

**Table 3:** Essential oils and volatile compounds modulating the antibiotic activity against *Klebsiella pneumoniae*.

Essential Oil/Volatile Compound	Vegetal Source	Major Constituent	*Klebsiella pneumoniae* Strain	Antibiotic	Method	Effect	Ref.
Peppermint EO	*Menthae folium* (*Mentha piperita* L., Lamiaceae, Peppermint)	-	NCTC 9633	Ciprofloxacin	CB (FICI = 0.68–0.90)	S	[2]
					CB (FICI = 1.40–2.24)	A	
Rosemary EO	*Rosmarini folium* (*Rosmarinus officinalis* L., Lamiaceae, Rosemary)	-	NCTC 9633	Ciprofloxacin	CB (FICI = 0.28–0.97)	S	
					CB (FICI = 1.03–1.07)	A	
Thyme EO	*Thymi herba* (*Thymus vulgaris* L., Lamiaceae, Thyme)	-	NCTC 9633	Ciprofloxacin	CB (FICI = 0.71–0.90)	S	
					CB (FICI = 1.10–1.40)	A	
Thyme EO	*Thymi herba* (*Thymus maroccanus* L., Lamiaceae, Za’ater/Azukeni)	Carvacrol (76.35%)	Clinical isolate	Ciprofloxacin	CB (FICI = 0.37)	S	[57]
				Gentamicin	CB (FICI = 0.50)	S	
				Pristinamycin	CB (FICI = 0.50)	S	
				Cefixime	CB (FICI = 1)	I	
Thyme EO	*Thymi herba* (*Thymus broussonetii* L., Lamiaceae, Za’atar Essaouiri)	Carvacrol (39.77%) Borneol (12.03%)		Ciprofloxacin	CB (FICI = 0.62)	PS	
				Gentamicin	CB (FICI = 0.62)	PS	
				Pristinamycin	CB (FICI = 0.50)	S	
				Cefixime	CB (FICI = 1)	I	
Lemon thyme EO	*Thymi herba* (*Thymus pulegioides* L., Lamiaceae, Lemon thyme/broad-leaved thyme)	Geraniol (66.59%)	ATCC 700603	Tetracycline	CB (FICI = 0.76–0.82)	S	[74]
					CB (FICI = 0.92–1.10)	Ad	
					CB (FICI = 1.16–1.28)	A	
				Streptomycin	CB (FICI = 0.32–0.87)	S	
					CB (FICI = 1.09)	Ad	
					CB (FICI = 1.16)	A	
				Chloramphenicol	CB (FICI = 0.32–0.87)	S	
					CB (FICI = 0.98–1.09)	Ad	
Savory EO	*Saturejae herba* (*Satureja kitaibelii* Wierzb. ex Heuff., Lamiaceae, Savory)	Geraniol (50.4%)	ATCC 700603	Chloramphenicol	CB (FICI = 0.21–0.43)	S	[60]
					CB (FICI = 0.54–0.98)	Ad	
					CB (FICI = 1.09)	I	
				Tetracycline	CB (FICI = 0.43)	S	
					CB (FICI = 0.56–0.98)	Ad	
					CB (FICI = 1.09)	I	
Thyme EO	*Thymi herba* (*Thymus saturejoides* Coss., Lamiaceae, Azoukni)	Carvacrol (25.3%) Borneol (19.7%)	Clinical isolate	Cefixime	CB (FICI = 0.75)	PS	[76]
		Carvacrol (26.5%) Borneol (20.1%)			CB (FICI = 0.75)	PS	
		Carvacrol (45.3%) Borneol (7.5%)			CB (FICI = 0.50)	S	
Sandarac EO	*Tetraclinis coni* (*Tetraclinis articulata* (Vahl.) Masters., Cupressaceae, Sandarac)	α-Campholenal (16.34%) *Trans*-Pinocarveol (15.45%) Verbenone (13.36%) *Cis*-Verbenol (12.36%)	CIP 8291	Amoxicillin	CB (FICI = 0.8)	PS	[75]
Tea tree EO	*Melaleucae folium* (*Melaleuca alternifolia*, (Maiden. and Betch.) Chee., Myrtaceae, Tea tree)	-	NCTC 9633	Ciprofloxacin	CB (FICI = 0.73–0.95)	S	[2]
					CB (FICI = 1.03–1.85)	A	
Geraniol	-		ATCC 700603	Chloramphenicol	CB (FICI = 0.32–0.87)	S	[60]
					CB (FICI = 0.98–1.09)	Ad	
				Tetracycline	CB (FICI = 0.76–0.80)	S	
					CB (FICI = 0.95–1.10)	Ad	
					CB (FICI = 1.25–1.66)	A	

A: antagonism; Ad: addition; I: indifference; PS: partial synergy; S: synergy; CB: checkerboard assay; FICI: fractional inhibitory concentration index.

**Table 4:** Essential oils and volatile compounds modulating the antibiotic activity against *Pseudomonas aeruginosa*.

Essential Oil/Volatile Compound	Vegetal Source	Major Constituent	*Pseudomonas aeruginosa* Strain	Antibiotic	Method	Effect	Ref.
Thyme EO	*Thymi herba* (*Thymus maroccanus* L., Lamiaceae, Za’ater/Azukeni)	Carvacrol (76.35%)	Clinical isolate	Ciprofloxacin	CB (FICI = 0.15)	S	[57]
				Gentamicin	CB (FICI = 0.18)	S	
				Pristinamycin	CB (FICI = 0.75)	PS	
				Cefixime	CB (FICI = 0.75)	PS	
Za’atar Essaouiri EO	*Thymi herba* (*Thymus broussonetii* L., Lamiaceae, Za’atar Essaouiri)	Carvacrol (39.77%) Borneol (12.03%)		Ciprofloxacin	CB (FICI = 0.14)	S	
				Gentamicin	CB (FICI = 0.28)	S	
				Pristinamycin	CB (FICI = 0.75)	PS	
				Cefixime	CB (FICI = 0.5)	S	
Lemon thyme EO	*Thymi herba* (*Thymus pulegioides* L., Lamiaceae, Lemon thyme/broad-leaved thyme)	Geraniol (66.59%)	ATCC 27853	Tetracycline	CB (FICI = 0.54–0.82)	S	[74]
					CB (FICI = 0.95–1.08)	Ad	
					CB (FICI = 1.16–1.28)	A	
				Streptomycin	CB (FICI = 1.20–2.00)	A	
				Chloramphenicol	CB (FICI = 0.43–0.87)	S	
					CB (FICI = 0.98–1.09)	Ad	
Savory EO	*Saturejae herba* (*Satureja kitaibelii* Wierzb. ex Heuff., Lamiaceae, Savory)	Geraniol (50.4%)	ATCC 27853	Tetracycline	CB (FICI = 0.54–0.82)	S	[60]
					CB (FICI = 0.96–1.10)	Ad	
					CB (FICI = 1.21–1.47)	A	
				Chloramphenicol	CB (FICI = 0.43–0.87)	S	
					CB (FICI = 0.98–1.09 )	Ad	
Thyme EO	*Thymi herba* (*Thymus saturejoides* Coss., Lamiaceae, Azoukni)	Carvacrol (25.3%) Borneol (19.7%)	ATCC 27853	Cefixime	CB (FICI = 0.28)	S	[76]
		Carvacrol (26.5%) Borneol (20.1%)			CB (FICI = 0.31)		
		Carvacrol (45.3%) Borneol (7.5%)			CB (FICI = 0.29)		
Sandarac EO	*Tetraclinis coni* (*Tetraclinis articulata* (Vahl.) Masters., Cupressaceae, Sandarac)	α-Campholenal (16.34%) *Trans*-Pinocarveol (15.45%) Verbenone (13.36%) *Cis*-Verbenol (12.36%)	CIPA 22	Amoxicillin	CB (FICI = 1.00)	Ad	[75]
Cidreira-do-mato/cidreira-brava EO	*Hyptis folium* (*Hyptis martiusii* Benth., Lamiaceae, Cidreira-do-mato/cidreira-brava)	Bicyclogermacrene (10.6%) *Trans*-Caryophyllene (9.2%) Caryophyllene oxide (7.4%) 1,8-Cineole (7%)	ATCC 15442	Gentamycin	IZGC (+18.9%)	S	[64]
					FR (–)	I	
				Amikacin	IZGC (+60%)	S	
					FR4-fold increase in MIC	A	
				Tobramycin	IZGC (+12.5%)	S	
				Neomycin	FR (–)	I	
				Kanamycin	FR (–)	I	
Marjoram EO	*Origani folium* (*Origanum majorana* L., Lamiaceae, Marjoram)	4-Terpineol (21.3%)	ATCC 9027	Piperacillin	IZDC (+41.2%)	S	[101]
				Cefepime	IZDC (+10%)	S	
				Meropenem	IZDC (+20.5%)	S	
				Gentamicin	IZDC (+31.03%)	S	
				Norfloxacin	IZDC (−0.03%)	A	
Sage EO	*Salviae folium* (*Salvia officinalis* L., Lamiaceae, Sage)	1,8-Cineole (29%)		Piperacillin	IZDC (+29.4%)	S	
				Cefepime	IZDC (−0.03%)	A	
				Meropenem	IZDC (+23.5%)	S	
				Gentamicin	IZDC (+13.7%)	S	
				Norfloxacin	IZDC (−0.16%)	A	
Thyme EO	*Thymi herba* (*Thymus vulgaris* L., Lamiaceae, Thyme)	Thymol (33.6%)		Piperacillin	IZDC (+147%)	S	
				Cefepime	IZDC (+53.3%)	S	
				Meropenem	IZDC (+52.9%)	S	
				Gentamicin	IZDC (+37.9%)	S	
Basil EO	*Basilici herba* (*Ocimum basilicum* L., Lamiaceae, Basil)	Linalool (55.2%)	ATCC 25853	Imipenem	CB (FICI = 0.75)	Ad	[99]
				Ciprofloxacin	CB (FICI = 1.03)	I	
			1662339	Imipenem	CB (FICI = 0.0625)	S	
				Ciprofloxacin	CB (FICI = 0.09)	S	
Alecrim-de-tabuleiro EO	*Lippiae folium* (*Lippia microphylla* Cham., Verbenaceae, Alecrim-de-tabuleiro)	1,8-Cineole (18.1%) *Z*-β-Ocimene (15.2%) Bicyclogermacrene (11.6%)	ATCC 15442	Gentamicin	IZGC (+47%)	S	[112]
				Tetracycline	IZGC (−14%)	A	
Creeping lantana EO	*Lantanae folium* (*Lantana montevidensis* Briq., Verbenaceae, Creeping lantana)	β-Caryophyllene (31.50%) Germacrene D (27.50%)	ATCC 15442	Gentamicin	IZGC (+12%)	S	[113]
				Amikacin	IZGC (+102%)		
Alecrim pimento EO	*Lippiae folium* (*Lippia sidoides* Cham., Verbenaceae, Alecrim pimento)	Thymol (84.9%)	ATCC 15442	Gentamycin	FR (4-fold reduction in MIC)	S	[102]
				Neomycin	FR (2-fold reduction in MIC)		
Alecrim-da-chapada EO	*Lippiae folium* (*Lippia gracilis* Schauer., Verbenaceae, Alecrim-da-chapada)	Thymol (44.4%) Carvacrol (22.2%)	ATCC 15442	Amikacin	IZGC	S	[107]
				Tobramycin	IZGC		
				Gentamycin	IZGC		
Candeeiro EO	*Vanillosmopsis* *cortex* (*Vanillosmopsis arborea* Baker., Asteraceae Candeeiro)	α-Bisabolol (80.43%)	ATCC 15442	Gentamycin	IZGC (+8.6%)	S	[108]
				Tetracycline	IZGC (−8.0%)	A	
				Tobramycin	IZGC (−18.0%)	A	
Immortelle EO	*Helichrysi flos* (*Helichrysum italicum* (Roth) G. Don, Asteraceae, Immortelle)	-	PAO1 PA124	Chloramphenicol	FR (16-fold reduction in MIC)	S	[37]
					FR (8-fold reduction in MIC)	S	
Canela de Cunha EO	*Crotonii folium* (*Croton zehntneri* Pax et Hoffm., Euphorbiaceae Canela de Cunha)	*Trans*-Anethole	ATCC 15442	Gentamycin	IZGC (+42.8%)	S	[109]
				Tetracycline	IZGC (0%)	I	
Limão-bravo EO	*Zanthoxylii folium* (*Zanthoxylum articulatum* Engler Rutaceae, Limão-bravo)	Viridiflorol (35.4%)	ATCC 15442	Gentamycin	IZGC (+43.8%)	S	[106]
				Tetracycline	IZGC (+9.6%)	Ad	
Eugenol	-		NCIM 5029	Ampicillin	CB (FICI < 0.50)	S	[115]
				Chloramphenicol			
				Erythromycin			
				Norfloxacin			
				Oxacillin			
				Penicillin			
				Polymyxin			
				Rifampicin			
				Erythromycin			
Thymol	**-**		ATCC 15442	Gentamycin	FR (4-fold reduction in MIC)	S	[102]
				Neomycin	FR (2-fold reduction in MIC)		
Geraniol	-		ATCC 27853	Chloramphenicol	CB (FICI = 0.54–0.87)	S	[60]
					CB (FICI = 0.92–1.09)	Ad	
				Tetracycline	CB (FICI = 0.76)	S	
					CB (FICI = 0.92–1.10)	Ad	
					CB (FICI = 1.16–1.47)	A	

A: antagonism; Ad: addition; I: indifference; PS: partial synergy; S: synergy; CB: checkerboard assay; FICI: fractional inhibitory concentration index; FR: fold reduction in MIC; MIC: minimum inhibitory concentration; IZGC (%): percentage change in inhibition zone diameter due to gaseous contact with essential oil/volatile component; IZDC (%): percentage change in inhibition zone diameter due to direct contact with essential oil/volatile component.

**Table 5:** Essential oils and volatile compounds modulating the antibiotic activity against other Gram-negative bacteria.

Essential Oil/Volatile Compound	Vegetal Source	Major Constituent	*Other Gram-negative* Bacteria	Antibiotic	Method	Effect	Ref.
***Enterobacter aerogenes*** **Strain**							
Thyme EO	*Thymi herba* (*Thymus maroccanus* L., Lamiaceae, Za’ater/Azukeni)	Carvacrol (76.35%)	ATCC 13048	Chloramphenicol	FR (4-fold reduction in MIC)	S	[52]
			EA27clinical isolate		FR (8-fold reduction in MIC)		
Thyme EO	*Thymi herba* (*Thymus broussonetii* L., Lamiaceae, Za’atar Essaouiri)	Carvacrol (39.77%) Borneol (12.03%)	ATCC 13048		FR (4-fold reduction in MIC)		
			EA27clinical isolate		FR (8-fold reduction in MIC)		
Immortelle EO	*Helichrysi flos* (*Helichrysum italicum* (Roth) G. Don, Asteraceae, Immortelle)	-	ATCC 13048	Chloramphenicol	FR (2-fold reduction in MIC)	S	[37]
			EAEP289	Chloramphenicol	FR (8-fold reduction in MIC)		
				Ampicillin	FR (–)	I	
				Penicillin	FR (–)		
				Norfloxacin	FR (2-fold reduction in MIC)	S	
			EAEP294	Chloramphenicol	FR (128-fold reduction in MIC )		
				Ampicillin	FR (~7300-fold reduction in MIC)		
				Penicillin	FR (~14600-fold reduction in MIC)		
				Norfloxacin	FR (~914-fold reduction in MIC)		
Eugenol	-		NCIM 5139	Ampicillin	CB (FICI < 0.50)	S	[115]
				Penicillin			
				Oxacillin			
				Erythromycin			
				Norfloxacin			
				Chloramphenicol			
				Polymyxin B			
				Tetracycline			
				Vancomycin			
				Rifampin			
***Enterobacter cloacae*** **Strain**							
Thyme EO	*Thymi herba* (*Thymus maroccanus* L. Lamiaceae, Za’ater/Azukeni)	Carvacrol (76.35%)	Clinical isolate	Ciprofloxacin	CB (FICI = 0.37)	S	[57]
				Gentamicin	CB (FICI = 0.19)		
				Pristinamycin	CB (FICI = 0.50)		
				Cefixime	CB (FICI = 1.00)	I	
Thyme EO	*Thymi herba* (*Thymus broussonetii* L. Lamiaceae, Za’atar Essaouiri)	Carvacrol (39.77%) Borneol (12.03%)		Ciprofloxacin	CB (FICI = 0.50)	S	
				Gentamicin	CB (FICI = 0.50)		
				Pristinamycin	CB (FICI = 0.50)		
				Cefixime	CB (FICI = 1.00)	I	
Alecrim pimento EO	*Lippiae folium* (*Lippia sidoides* Cham. Verbenaceae, Alecrim pimento)	Thymol (84.9%)	ATCC 23355	Gentamicin	FR (–)	I	[102]
				Neomycin			
				Penicillin G			
				Ceftriaxone			
Thymol	-		ATCC 23355	Gentamicin	FR (–)	I	
				Neomycin			
				Penicillin G			
				Ceftriaxone			
***Proteus vulgaris*** **Strain**							
α-Bisabolol	Candeeiro EO Candeeiro stem *Vanillosmopsis arborea* Baker. Asteraceae	-	ATCC 13315	Gentamycin	IZGC (+4%)	I	[108]
				Tetracycline	IZGC (−51.72%)	A	
				Tobramycin	IZGC (−53%)	A	
Eugenol	-		NCIM 2813	Ampicillin	CB (FICI < 0.50)	S	[115]
				Penicillin			
				Oxacillin			
				Erythromycin			
				Norfloxacin			
				Chloramphenicol			
				Polymyxin B			
				Tetracycline			
				Vancomycin			
				Rifampin			
***Salmonella typhimurium*** **Strain**							
Thyme EO	*Thymi herba* (*Thymus maroccanus* L., Lamiaceae, Za’ater/Azukeni)	Carvacrol (76.35%)	Clinical isolate	Ciprofloxacin	CB (FICI = 0.37)	S	[57]
				Gentamicin	CB (FICI = 0.75)	PS	
				Pristinamycin	CB (FICI = 0.75)		
				Cefixime	CB (FICI = 0.18)	S	
Thyme EO	*Thymi herba* (*Thymus broussonetii* L., Lamiaceae, Za’atar Essaouiri)	Carvacrol (39.77%) Borneol (12.03%)		Ciprofloxacin	CB (FICI = 0.56)	PS	
				Gentamicin	CB (FICI = 0.62)		
				Pristinamycin	CB (FICI = 0.50)	S	
				Cefixime	CB (FICI = 0.18)		
Carvacrol	-		Clinical isolate	Nalidixic acid	CB (FICI = 0.31)	S	[124]
			SGI1	Ampicillin	CB (FICI = 0.25)		[66]
				Penicillin	CB (FICI = 0.37)		
				Tetracycline	CB (FICI = 0.18)		
				Erythromycin	CB (FICI = 0.25)		
				Bacitracin	CB (FICI = 0.25)		
				Novobiocin	CB (FICI = 0.37)		
Eugenol	-		SGI1	Ampicillin	CB (FICI > 0.5)	I	
				Penicillin	CB (FICI > 0.5)		
				Tetracycline	CB (FICI = 0.22)	S	
				Erythromycin	CB (FICI = 0.63)	I	
				Bacitracin	CB (FICI > 0.5)		
				Novobiocin	CB (FICI = 0.40)	S	
Eugenol	-		NCIM 2501	Ampicillin	CB (FICI < 0.50)	S	[115]
				Penicillin			
				Oxacillin			
				Erythromycin			
				Norfloxacin			
				Chloramphenicol			
				Polymyxin B			
				Tetracycline			
				Vancomycin			
				Rifampin			
Thymol	-		SGI1	Ampicillin	CB (FICI = 0.12)	S	[66]
				Penicillin	CB (FICI = 0.13)		
				Tetracycline	CB (FICI = 0.10)		
				Erythromycin	CB (FICI = 0.25)		
				Bacitracin	CB (FICI = 0.15)		
				Novobiocin	CB (FICI = 0.37)		
Cinnamaldehyde	-		SGI1	Ampicillin	CB (FICI = 0.25)	S	
				Penicillin	CB (FICI = 0.63)	I	
				Tetracycline	CB (FICI = 0.37)		
				Erythromycin	CB (FICI = 0.24)		
				Bacitracin	CB (FICI = 0.24)		
				Novobiocin	CB (FICI = 0.24)		
Allyl isothiocyanate	-		SGI1	Ampicillin	CB (FICI = 0.35)	S	[66]
				Penicillin	CB (FICI = 0.63)	I	
				Tetracycline	CB (FICI = 0.73)		
				Erythromycin	CB (FICI = 0.48)	S	
				Bacitracin	CB (FICI = 0.50)		
				Novobiocin	CB (FICI = 1.00)	I	

A: antagonism; I: indifference; PS: partial synergy; S: synergy; CB: checkerboard assay; FICI: fractional inhibitory concentration index; FR: fold reduction in MIC; MIC: minimum inhibitory concentration; IZGC (%): percentage change in inhibition zone diameter due to gaseous contact with essential oil/volatile component.
